# Stem Cell Therapy for the Treatment of Myocardial Infarction: How Far Are We Now?

**DOI:** 10.7759/cureus.17022

**Published:** 2021-08-09

**Authors:** Rinky A Botleroo, Renu Bhandari, Rowan Ahmed, Roaa Kareem, Mallika Gyawali, Nanditha Venkatesan, Opemipo D Ogeyingbo, Abeer O Elshaikh

**Affiliations:** 1 Internal Medicine, California Institute of Behavioral Neurosciences & Psychology, Fairfield, USA; 2 Internal Medicine, Manipal College of Medical Sciences, Pokhara, NPL; 3 Internal Medicine, All India Institute of Medical Sciences, Raipur, IND; 4 Internal Medicine, Saint James School of Medicine, Park Ridge, USA; 5 Public Health, Walden University, Minneapolis, USA

**Keywords:** stem cells, myocardial infarction, stem cell therapy, bone marrow mononuclear cells, stemi, stem cell transplantation

## Abstract

Myocardial infarction is one of the leading causes of death worldwide. Poor functional recovery of the myocardium is noticed after an event of myocardial infarction. Researchers and clinicians around the world have been engaged to regenerate the damaged human heart for a long time. Stem cell therapy is an exciting newer therapy to treat cardiovascular diseases.

Various types of stem cells have been used to revive the damaged myocardium after myocardial infarction, and they have overall demonstrated safety and moderate efficacy. The specific mechanisms by which these cells help in improving cardiac function are still not completely known. There is growing evidence that intracoronary bone marrow cell transplantation in patients with myocardial infarction beneficially affects the remodeling of the damaged myocardium.

Our systematic review article aims to assess the effects and the future of stem cell therapy in patients with myocardial Infarction. We searched articles in PubMed, ScienceDirect, and Google Scholar. Thirty-one studies that included 2171 patients in total were analyzed. Most of these studies showed stem cell therapy is safe and well tolerated in patients, and modest improvements are seen in left ventricular functions with no major adverse effects. However, some studies showed no positive and clinically significant outcomes. So, more high-quality studies on a larger scale are required to support and confirm its efficacy in remodeling damaged myocardium after myocardial infarction. We should also perform studies to determine the timing of cell delivery that is best suited for stem cell therapy.

## Introduction and background

One of the most common causes of morbidity and mortality globally is cardiovascular disease [1]. The one-year mortality is approximately 13% and the five-year prognosis for patients with heart failure is 50%, even though there has been tremendous advancement in the treatment of acute myocardial infarction (MI) [1].

The presence of any obstruction in the coronary arteries gives rise to acute myocardial ischemia [1]. Rupture of plaques, fissuring, or formation of any superimposed thrombus may be responsible for this obstruction formation [1]. Although there have been major advancements in the management of acute Myocardial Infarction including fibrinolysis and rapid revascularization, the prognosis remains poor due to the lack of self-repairing of the already damaged myocardium, which may result in complications like heart failure [1]. 

There are multiple methods to repair the damaged heart that include cell transplantation, gene therapy, stimulating innate repair pathways, direct reprogramming of cells, cardiac tissue engineering, and biomaterial delivery [2]. Among these, the most accepted strategy for heart repair is the delivery of exogenous cells [2]. Almost every cell type we can think of, such as skeletal myoblasts to pluripotent stem cells and their derivatives has been transplanted into the injured myocardium [2].

Stem cells are unspecialized immature cells that can divide and replicate themselves throughout the entire life of an organism [3]. Skeletal myoblasts (satellite cells) are classically the stem cell population within the non-cardiac musculature [4]. There are 2% to 7% improvements in ejection fractions (EF) with the administration of adult bone marrow cells (BMC) [4]. The exact mechanisms of improvement of damaged heart function by cell therapy are unclear, but it is assumed that the paracrine effect plays a central role [5]. Transplanted mesenchymal stem cells (MSCs) can engraft and differentiate into cardiomyocyte-like and endothelial cells and recruit endogenous cardiac stem cells [6]. 

As the viability and function of autologous adult stem cells decline with age, especially in patients with MI, alternative sources of stem cells such as Wharton’s jelly-derived mesenchymal stem cells (WJ-MSCs), cardiac progenitor cells can also be used [6]. Isolating and expanding resident cardiac progenitor cells present in the adult myocardium cells is a tough task. However, these are more beneficial than the other stem cell types because they are likely predestined to cardiovascular fate [5].

**Figure 1:**
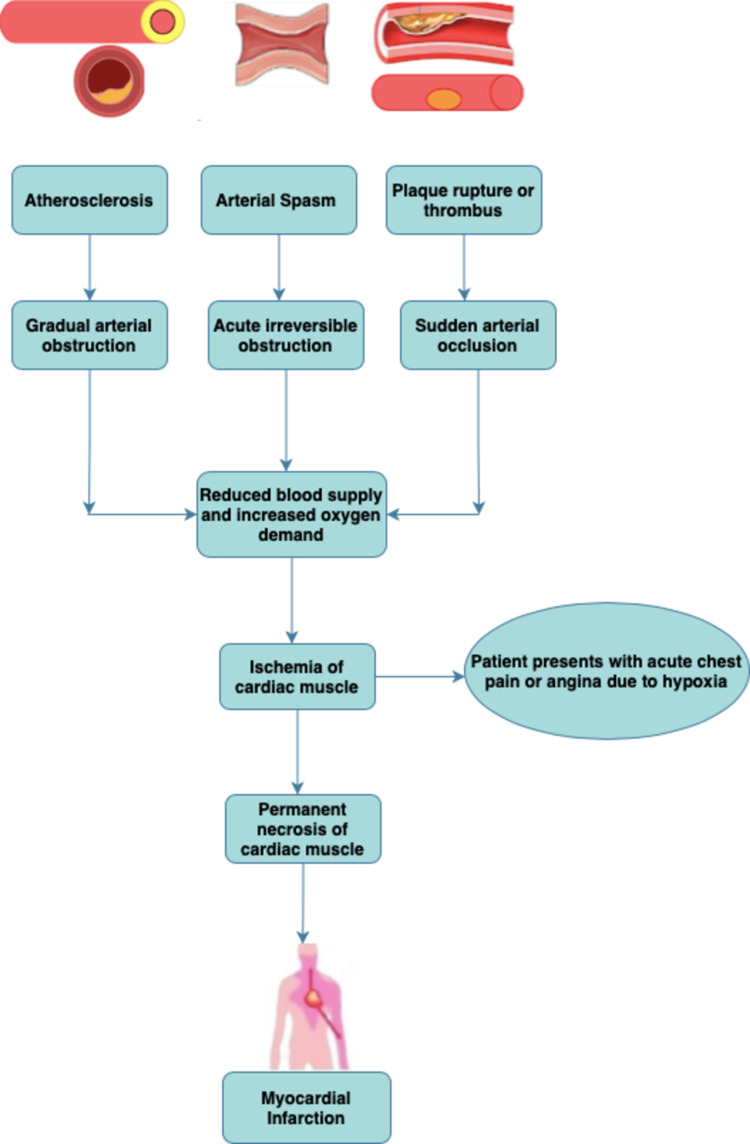
Pathophysiology of myocardial infarction

The first-ever encouraging study showing positive outcomes in MI patients with stem cell therapy was published by Strauer et al. in 2002, many other trials have been conducted since then [7]. The main objective of our article is to evaluate the safety and effects of transplanting stem cells in patients with acute myocardial infarction. Figure 1 given below illustrates the pathophysiology of MI.

## Review

Methods

Study Protocol

We implemented Preferred Reporting Items for Systematic Review and Meta-Analyses (PRISMA) 2020 Guidelines in our study process. 

Sources of Data and Search Strategy

Articles were searched from three databases PubMed, Science Direct, and Google Scholar using specific keywords related to the research topic. The keywords used are as follows: myocardial infarction, ST-elevation myocardial infarction, non-ST-elevation myocardial infarction, stem cell transplantation, mononuclear bone marrow cell transplantation, adult population.

The Medical Subject Headings (MeSH) is the National Library of Medicine (NLM) controlled vocabulary thesaurus that we specifically used to search articles in the PubMed database. We performed a search using a combination of MeSH terms and text words given below. The final MeSH search strategy used was as follows: 

Myocardial Infarction OR ST-Elevation Myocardial Infarction OR Non-ST-Elevation Myocardial Infarction OR Acute Myocardial Infarction OR ( "Myocardial Infarction/drug therapy"[Mesh] OR "Myocardial Infarction/mortality"[Mesh] OR "Myocardial Infarction/prevention and control"[Mesh] OR "Myocardial Infarction/therapy"[Mesh] ) AND Stem Cell Transplantation OR Stem cell therapy OR mesenchymal stem cell OR Progenitor Cell OR mononuclear bone marrow cell transplantation OR ( "Stem Cell Transplantation/instrumentation"[Mesh] OR "Stem Cell Transplantation/methods"[Mesh] OR "Stem Cell Transplantation/mortality"[Mesh] OR "Stem Cell Transplantation/therapeutic use"[Mesh] OR "Stem Cell Transplantation/therapy"[Mesh] )AND Adult.

Other databases and the keywords used for the search are mentioned in Table 1.

**Table 1:** Databases and search results

Databases	Keywords used for the search	Search results	
		Initial results	Timeframe 2011-2021
PubMed	The final MeSH search strategy as mentioned above	107,272	57,904
ScienceDirect	Myocardial Infarction and Stem cell therapy	22,211	12,640
Google Scholar	"Myocardial infarction" and "stem cell therapy" and "mononuclear bone marrow cell transplantation"	1,300	547

Inclusion and Exclusion Criteria

We only included the articles published in the English language, which were human studies and clinical trials. We selected articles published from 2011-2021. The inclusion criteria were: (1) Patients diagnosed with myocardial infarction; (2) Patients who received stem cell therapy after myocardial infarction; (3) Age of the patients 19 and above; (4) Both male and female patients were selected. Exclusion criteria were studies on animals, reviews, or studies for which the full text was unavailable or only abstracts were available. We did not include gray literature.

Risk and Quality Assessment

Two reviewers independently (RAB and RB) extracted and evaluated the quality of the included 31 studies. Revised Cochrane’s risk of bias assessment tool was used for randomized controlled trials (RCTs) and clinical trials.

Data Extraction

Two reviewers (RAB and RB) separately extracted relevant data from included 31 studies using standard data extraction forms and data was extracted under the following headings: name of the author, country of the study, the name of the journal where it was published, year of publication, study design, the title of the study, sample size, patient characteristics, size of the treatment group and control group, follow-up period, and outcome of the study.

Results

**Figure 2:**
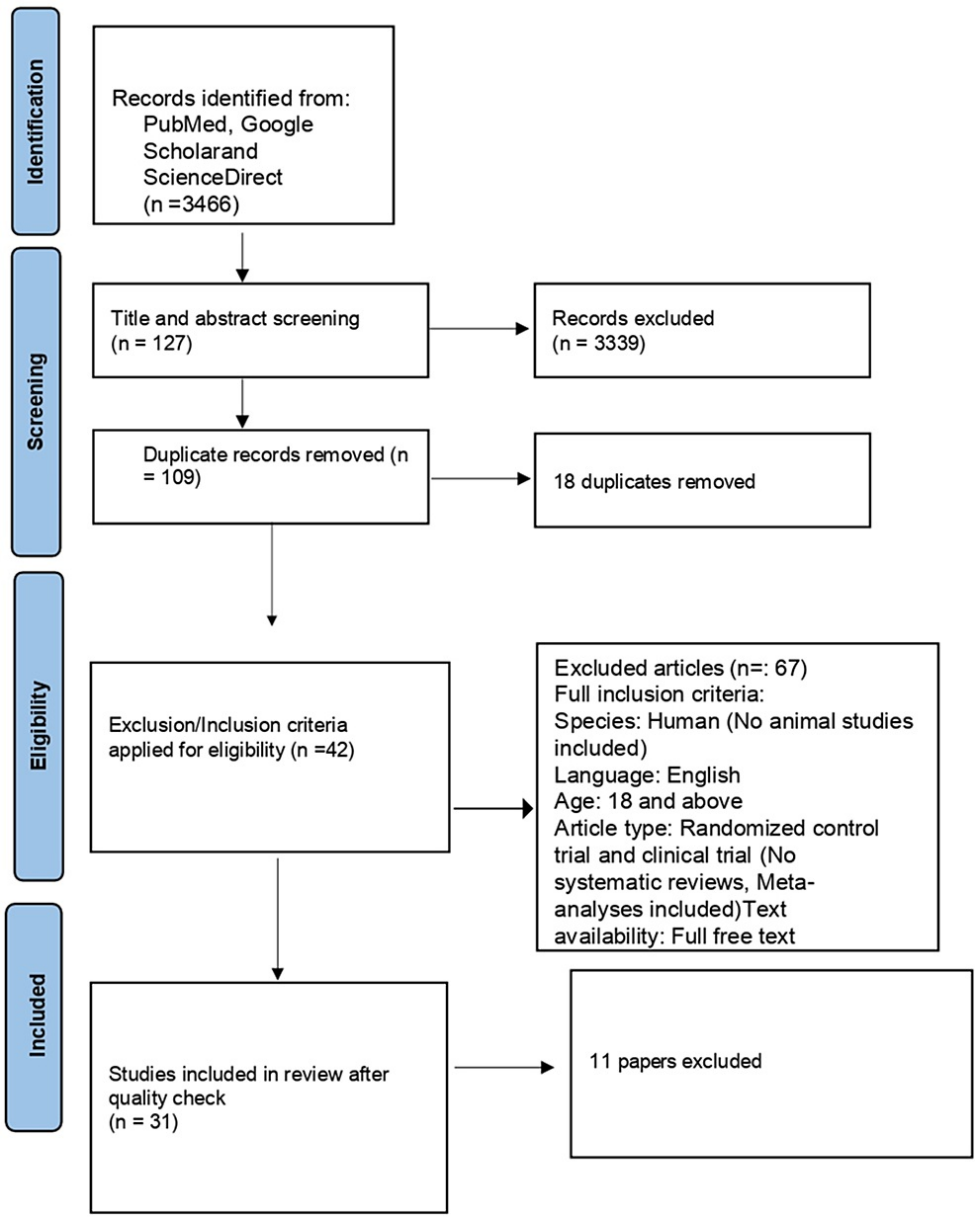
PRISMA flow diagram PRISMA: Preferred Reporting Items for Systematic Review and Meta-Analyses

A total of 2650 articles from PubMed, 221 articles from ScienceDirect, and 547 articles from Google Scholar were collected using the search strategy we have mentioned in the method section and were then screened based on the title and abstract related to our study. We also removed the duplicates. Then, we filtered out a few papers based on the eligibility criteria and availability of full text. In the end, only 31 items were included, and these articles were checked for quality based on their study characteristics. A complete Preferred Reporting Items for Systematic Review and Meta-Analyses (PRISMA) flow diagram is given below in Figure 2. 

Study Characteristics

Our systematic review includes patients from 31 studies. Table 2 shows the characteristics of the included studies and the outcomes of the studies.

**Table 2:** Characteristics and outcomes of the included studies STEMI = ST-Elevation Myocardial Infarction, CAG = Coronary Angiography, PCI = Percutaneous Coronary Intervention, PPCI = Primary Percutaneous Coronary Intervention, LVEF = Left Ventricular Ejection Fraction, CABG = Coronary artery bypass grafting, CK = Creatine Kinase, H/o = History of, MI = Myocardial Infarction, CCT = Controlled Clinical Trial, CT = Clinical Trial, MNC = Mononuclear stem cells, CCTA = Coronary Computed Tomography Angiography, RCT = Randomized Controlled Trial, BMMNC = Bone Marrow Mononuclear Cell, BNP = B-type natriuretic peptide, WMSI = Wall Motion Score Index, N-BMC = Normoxia Bone Marrow Mononuclear Cells, HP-BMC = Hypoxia Preconditioned Bone Marrow Mononuclear Cells, ECG = Electrocardiogram, SPECT = Single-Photon Emission Computed Tomography, LV = Left Ventricle, MRI = Magnetic Resonance Imaging, CT scan = Computed Tomography scan, F-18-FDG-PET = F-18-Fluorodeoxyglucose Positron Emission Computed Tomography, 99mTc-SPECT= 99mTc-sestamibi Single-Photon Emission Computed Tomography, G-CSF = Granulocyte Colony-Stimulating Factor, MSCs = Mesenchymal Stem Cells, WJ-MSC = Wharton’s jelly-derived Mesenchymal Stem Cells, CDC = Cardio sphere-derived autologous stem cell, LVEDD = End-Diastolic Dimension of the Left Ventricle, LVESV = End-Systolic Volume of the Left Ventricle, LVEDV = End-Diastolic Volume of the Left Ventricle, SPCs = Stem/ Progenitor Cells, LIN- = Lineage-negative, CADUCEUS = CArdiosphere-Derived aUtologous stem CElls to reverse ventricUlar dySfunction, TIME trial = Timing in Myocardial Infarction Evaluation trial

Author	Country/Year	Study design	Patient characteristics	Sample size	Treatment group	Control group	Follow-up period	Outcome
Benedek et al. [8]	Romania / 2014	CCT	Adult with h/o STEMI and PPCI and abnormalities in wall motion and less than 50% stenosis	18	Autologous MNC = 9	Placebo = 9	Four years with clinical examinations, ECG, Echocardiography, 64-slice CCTA	A small improvement in EF and the plaque burden is lower in coronary segments treated with stem cells
Alestalo et al. [9]	Finland / 2015	Double-blinded RCT	H/o STEMI, < 75 years, hemodynamically stable and no cardiogenic shock or rescue PCI/CABG	26	BMMNC = 14	Placebo = 12	Cytokines after four days and LV angiogram after six months	A balancing effect between the anti-inflammatory and proinflammatory cytokine BMMNC group at day four
Bozdag-Turan et al. [10]	Germany / 2012	CCT	18–80 years with h/o STEMI, absence of co-morbidities, cancer, and active bleeding or trauma in the last two months	24	BMC = 12	Placebo = 12	Six months with left ventriculography	Infarct size and BNP level decreased, and global EF and infarct wall movement velocity were increased in the stem cell group
Choudry et al. [11]	5 centers in Europe [United Kingdom (3); Switzerland (1); Denmark (1)] / 2016	Double-blinded RCT	Acute anterior MI with anterior wall motion abnormality and h/o PPCI	100	BMMNC = 55	Placebo = 45	One year with cardiac MRI and Echocardiography	Small non-significant improvement in LVEF
Duan et al. [12]	China / 2015	RCT	H/o MI, < 75 years with planned CABG for triple-vessel disease, LVEF < 30%, and no aneurysm or valvular diseases	42	CABG + BMMNC = 24	Only CABG = 18	One year with Echocardiography	Improvement in left ventricular functions in the treatment group
Gao et al. [6]	China / 2015	Double-blinded RCT	18-80 years with a h/o STEMI and reperfusion with stent implantation and LV local wall-motion abnormality. CK > three-fold the upper limit of the normal	116	WJ-MSC = 58	Placebo = 58	18 months with F-18-FDG-PET and 99mTc-SPECT and two-dimensional Echocardiography	LVEF significantly increased and LVESV and LVEDV greatly decreased in the treatment group
Hu et al. [13]	China / 2015	RCT	18-75 years old with acute STEMI and PPCI with stent implantation or thrombolysis and LV local wall motion abnormality	36	N-BMCs = 11, HP-BMCs = 11	Standard therapy = 14	Six and 12 months	Improvement in changes of LVEDV, LVESV, and WMSI in the HP-BMC group. Myocardial perfusion defect ratio was reduced in HP-BMCs and N-BMC groups at six months
Huan et al. [14]	China / 2015	CT	18-75 years old with a h/o acute STEMI and treatment with PCI, LVEF < 50%	104	Group A = BMMNC within two hours after PCI = 27, Group B = three-seven days after PCI = 26, Group C = seven to 30 days after PCI = 26	Placebo = 25 patients	Six months with angiography. SPECT and Echocardiography at six and 12 months	Effects of cell therapy given within 24 hours are the same as to given three-seven days after PPCI
Kim et al. [15]	South Korea / 2018	RCT	STEMI	26	BMMNC = 14	Placebo = 12	Four and 12-months with SPECT	Increase in the LVEF from baseline to the fourth month and twelfth month in the bone marrow mesenchymal stem cells group.
Lee et al. [16]	Korea / 2014	CT	18-70 years old with STEMI	69	MSC = 33	Placebo = 36	Six months with SPECT	Safe with modest improvement in LVEF
Makkar et al. [17]	USA / 2012	CT	H/o two to four weeks of MI and LVEF = 25–45%	25	Cardio sphere-derived autologous stem cells = 17	Standard therapy = 8	Six months with MRI	Scar mass was reduced, increase in viable heart mass and regional contractility in the CDC group. LVEDV, LVESV, and LVEF were the same in the two groups
Malliaras et al. [18]	USA / 2014	RCT	Patients of CADUCEUS trial were followed up for a year	25	Cardio sphere-derived autologous stem cells = 17	Standard therapy = 8	One year with MRI	Scar size reduced, increased viable myocardium, and improved regional function of infarcted myocardium
Micheu et al. [19]	Romania / 2015	CT	18-81 years old with STEMI & h/o angioplasty with stent implantation, LVEF < 40%.	18	Autologous BMCs = 7	Standard therapy = 11	Six months with clinical examination, Echocardiography, 24 hours ECG	Safe and LVEF was increased
Moccetti et al. [20]	Switzerland / 2012	CT	Acute anterior STEMI treated by PPCI and LVEF < 50%.	60	Autologous BMMNC = 23	Standard therapy = 37	Five years with Echocardiography	Safe and LV function improved
Moreira et al. [21]	Brazil / 2011	RCT	18-80 years old with h/o MI and reperfusion and involving more than 10% of the LV	30	BMMNC via anterograde intra-arterial coronary (IAC) delivery = 14, BMMNC via retrograde intravenous coronary (IVC) delivery = 10	Placebo = 6	Cardiac MRI was performed before cell injection	The retrograde approach to deliver stem cells was safe and cell retention by cardiac tissue is more in the anterograde approach
Nair et al. [22]	India / 2015	RCT	Anterior MI and LVEF = 20-50%, 20-65 years with h/o CAG between one to three weeks	250	Stem cell therapy + standard care = 125	Standard care = 125	Six months with Echocardiography	Safe, but not clinically significant
Naseri et al. [23]	Iran / 2018	RCT	18-75 years old with a h/o acute MI infarction, eligible for elective CABG	77	CD133 (+) = 21, MNC = 30	Placebo = 26	Six and 18 months after CABG with SPECT	Significant differences were seen between the MNC and placebo groups in LVEF and a decrease in the LV thickening
Nicolau et al. [24]	Brazil / 2018	RCT	30-80 years, LVEF ≤ 50%, and regional dysfunction in the infarct-related area	120	BMMNC = 66	Placebo = 55	Six months with MRI	No significant effects
Peregud-Pogorzelska et al. [25]	Poland / 2020	CCT not randomized	<65 years old with first MI and EF ≤ 45%	34	Standard therapy + autologous BM-derived LIN- SPCs = 15	Only standard therapy = 19	One, three, six months, and one year with Echocardiography	Safe and > 10% improvement in LVEF is noticed at 12 months
Quyyumi et al. [26]	USA / 2017	RCT	STEMI with a stent and LVEF ≤ 48% and ≥ four days post stent	161	Intracoronary infusion of autologous CD34 (+) cell = 78	Placebo = 83	Six months with SPECT	Safe
Rodrigo et al. [27]	Netherlands / 2013	CCT	First acute STEMI treated with PPCI and maximum CK level was > 1,600 U/L	54	MSC = 9	Standard therapy = 45 matched but nonrandomized patients	Three, six months, one year, four-five years with Echocardiography, Holter, and clinical examination	Improvements in LV function but not significantly different when compared to controls
Roncalli et al. [28]	France / 2011	RCT	Acute MI and successful reperfusion with LVEF ≤ 45%, age 18–75 years	101	BMMNC = 52	Placebo = 49	Three months with MRI, Echocardiography, and SPECT	Multivariate analysis shows improvement of myocardial viability than univariate analysis
San Roman et al. [29]	Spain / 2015	RCT	Adult, acute MI with PPCI or post-fibrinolysis PCI and rapamycin drug-eluting stent implantation	120	BMMNC = 30, GCSF = 30, G-CSF + cells = 29	Standard therapy = 31	12 months with cardiac MRI	Not many differences among the four groups
Shah et al. [3]	India / 2014	CT	30-70 years old, acute MI with PCI	19	Autologous BMCS = 12	Standard therapy = 7	24 months with Echocardiography, ECG, Holter monitoring	Increase in LVEF with LV function improvements in stem cell group
Srimahachota et al. [30]	Thailand / 2011	RCT	H/o STEMI with LVEF < 50% and PCI	23	Autologous BMCs = 11	Standard therapy = 12	Six months with cardiac MRI	Symptoms improved than baseline, but not many significant changes were noticed in the two groups
Sürder et al. [31]	Switzerland / 2013	RCT	Acute MI	200	BMMNC five-seven days after STEMI = 66, BMMNC three to four weeks after STEMI = 67	Standard therapy = 67	Four months with cardiac MRI	No significant improvements
Traverse et al. [32]	USA / 2012	RCT	MI and PCI, LVEF < 45%	120	BMMNC at day three or day seven randomly = 79	Placebo = 41	Six months with cardiac MRI	No significant improvement
Traverse et al. [33]	USA / 2011	RCT	Acute MI and PCI, LVEF < 45%	87	BMMNC after two to three weeks of MI = 58	Placebo = 29	Six months with cardiac MRI	No significant improvement
Traverse et al. [34]	USA / 2018	RCT	Patients of TIME trial, acute MI and PCI, LVEF < 45%	120	BMMNC at day three or day seven randomly = 79. 58 patients were followed up	Placebo = 41, 27 patients were followed up	Two years with cardiac MRI	No significant improvement
Turan et al. [35]	Germany / 2011	RCT	18-80 years old with MI and LV dysfunction	56	BMMNC = 38	Placebo = 18	Three, six months, and one year with left ventriculography	Decrease in infarct size but an increase of global EF and infarct wall movement velocity in stem cell group
Yerebakan et al. [36]	Germany / 2011	RCT	MI at least 14 days before admission and LV akinesia with an indication for CABG	55	Intramyocardial CD133 (+) BMCs + CABG = 35	Only CABG = 20	18 months with 24-hour Holter monitoring, echocardiography, MRI, and CT scan	Intramyocardial stem cell therapy was tolerable but did not have significant improvements

Discussion

After an acute myocardial infarction, patients usually suffer from left ventricular remodeling even after having successful revascularization [8]. Remodeling of the heart means changes in the size, shape, structure, and function of the cardiac muscles [37]. Our systematic review had 31 studies and 2171 patients. We observed the effectiveness of stem cells in an injured heart muscle after myocardial infarction.

Effect of Stem Cell Therapy on Heart Function After Myocardial Infarction

Benedek et al. conducted a controlled clinical trial (CCT). They included 18 patients in this trial, out of which nine patients received autologous bone marrow-derived mononuclear cells (BMMNC) [8]. On follow-up after four years, this study showed a slight improvement in ejection fraction (EF) in the stem cell group, the number of coronary plaques in segments infused with stem cell vs placebo group was ten vs twenty-one, calcium scoring in stem cell group vs placebo group was 295 vs 796, plaques creating > 50% stenosis in stem cell group vs placebo group were two vs eight and the plaque burden was much lower in coronary segments treated with stem cells [8].

Bozdag-Turan et al. conducted a prospective nonrandomized CCT in 24 patients in which he noticed a reduction in infarct size (p < 0.001), an increase in global EF (p = 0.003), and an increase in infarct wall movement velocity. Additionally, B-type natriuretic peptide (BNP) level also decreased in the stem cell group (p < 0.001) [10].

The clinical trial (CT) performed in 19 patients by Shah et al. demonstrated 12 patients who had received stem cell therapy, their echocardiography showed an increase in left ventricular ejection fraction (LVEF) from baseline at six months (3.8%) which was sustained at two years (1.63% increase), whereas in the control group LVEF was initially increased by 1.5% but at follow-up, in two years LVEF was decreased by 7.3% compared to the baseline [3].

Kim et al. concluded in their randomized clinical trial (RCT) of 26 patients that there was some improvement in LVEF [15]. Turan et al. in their RCT of 56 patients described there was a decrease in infarct size but an increase of global EF and infarct wall movement velocity in the stem cell group compared to the control group [35]. Similarly, Naseri et al. in their RCT of 77 patients noticed significant differences between the stem cell groups and placebo groups in LVEF and a decrease in the left ventricular (LV) thickening [23].

Gao et al. had 58 patients in their treatment group who had received 6 × 10^6^ Wharton's Jelly-derived mesenchymal stem cells (WJ-MSC) dispersed in 10 mL heparinized saline and 58 patients on the control arm who received placebo [6]. 18 months later, follow-up revealed LVEF in the WJ-MSC group significantly increased in comparison to the placebo group. Also, left ventricular end-systolic and end-diastolic volumes were greatly decreased in the WJ-MSC group [6].

In the non-randomized CCT of Peregud-Pogorzelska et al. in 34 patients, they found that stem cell therapy is safe and 60% of patients from the bone marrow-derived lineage negative (LIN-) stem/progenitor cell group showed about > 10% improvement in LVEF after a year with no signs of unfavorable remodeling of the left ventricle (LV) [25]. Similarly, one RCT conducted by Quyyumi et al. on 161 patients, among which 78 patients received an intracoronary infusion of autologous CD34 (+) cell (CLBS10) (cell therapy 10, Caladrius Biosciences Inc, Basking Ridge, NJ) revealed that stem cell therapy was safe and at one year, 3.6% and 0% deaths were observed in the control and treatment group, respectively [26].

Additionally, the clinical trials conducted by Lee et al. and Micheu et al. found stem cell therapy is safe [16,19]. 

Hu et al. in their RCT included 36 patients out of which 22 patients in the treatment arm either received normoxia-bone marrow cells (N-BMCs) or hypoxia-preconditioned bone marrow cells (HP-BMCs) and 14 patients received standard therapy [13]. There was an improvement in changes of left ventricular end-diastolic volume (LVEDV) and left ventricular end-systolic volume (LVESV) in HP-BMC group than N-BMC or control group (P < 0.05), wall motion score index (WMSI) got better in HP-BMCs and N-BMC group (P<0.050), but not in the control group [13]. Additionally, the myocardial perfusion defect ratio was reduced in HP-BMCs and N-BMC groups at six months compared with baseline [13].

Makkar et al. conducted a CT in 25 patients, among which 17 patients in the control group were given cardio sphere-derived autologous stem cells; they showed at follow-up after six months [17] and one year [18] that the scar size was reduced, myocardial viability was increased along with the improved regional function of the damaged myocardium. 

Roncalli et al. in their RCT included 52 patients receiving BMMNC and 49 patients receiving placebo [28]. Myocardial viability improved in 16/47 (34%) patients in the treatment arm compared to 7/43 (16%) in the control group (P = 0.06) and the number of non-viable segments becoming viable was 1.2 ± 1.5 in the BMMNC group and 0.8 ± 1.1 in the control group (P = 0.13) [28]. At three months follow-up, the multivariate analysis showed improvement of myocardial viability than the univariate analysis (P = 0.03) [28]. It also revealed that active smoking has a significant adverse effect (P = 0.04), and a positive trend for microvascular obstruction (P = 0.07) was observed as well [28]. 

Meanwhile, the double-blinded RCT of Alestalo et al. with 26 patients (14 receiving BMMNC and 12 receiving placebo) observed a harmonizing effect between the anti-inflammatory and pro-inflammatory cytokines in BMMNC treated ST-elevation myocardial infarction (STEMI) patients on day four [9]. The inflammation process of myocardial infarction (MI) was affected by this balancing effect, and it helped in remodeling and repair of the damaged heart muscles after an episode of acute MI [9].

In contrast, the double-blinded RCT of Choudry et al. in 100 patients showed although LVEF was increased compared with the baseline in both treatment and control groups, there was not much difference between the two groups (2.2%; 95% confidence interval, CI: −0.5 to 5.0; P = 0.10) at one-year [11]. 

The RCT conducted by Nair et al. in 250 patients revealed, even though it is safe, stem cell therapy has no benefit in STEMI [22]. The number of patients in this study receiving the stem cell therapy deviated from 125 to 71 patients and the follow-up period was relatively short, which might have affected the outcome [22].

Similarly, Nicolau et al. in their RCT of 120 patients found intracoronary infusion of autologous bone marrow-derived mononuclear cells (BMMC) to STEMI patients did not improve LV function or decrease scar size [24]. This study did not have a core cell-processing laboratory, there was an unbalanced enrollment by the centers, and they used LVEF as the endpoint which may not be the most suitable endpoint to investigate the effect of cell infusion due to its constant changes in the acute phase [24]. These all factors had influenced the result.

San Roman et al. in their RCT divided their study population into four groups which include one group of 30 patients receiving bone marrow mononuclear cells, 30 patients assigned to granulocyte colony-stimulating factor (G-CSF), 29 patients receiving G-CSF + cells, and a placebo group of 31 patients receiving standard therapy [29]. Patients treated with any of these stem cell approaches experienced similar changes in LVEF and LVESV when compared to the control group, with a small but significant reduction in infarct area (p = 0.038) [29]. One year later, cardiac magnetic resonance imaging (MRI) did not show much difference in these four groups [29]. But it was an open-labeled study and the study population was small which may have had an impact on the result of the study [29].

Srimahachota et al. concluded in their RCT in 23 patients that, stem cell therapy is safe but no improvement in LVEF can be described from the study [30]. The authors described a few reasons for not having a positive outcome such as the BMMNC cannot maintain at the infarcted area and a very few BMMNC remained at heart [30]. Also, cytokines may be needed to integrate the stem cells in the affected heart area to initiate the cells to trans-differentiate to cardiac myocyte, and the best cell type and timing for stem cell infusion is not yet known [30].

Similarly, Sürder et al. in their RCT of 200 patients [31] and Traverse et al. in their RCT of 120 patients explained that they did not notice any improvement in LV function in the stem cell group [32,34]. Traverse et al. demonstrated the use of cardiac MRI led to greater dropout of patients during the follow-up period because of device implantation in patients with more severe LV dysfunction which negatively impacted the outcome of their study [34].

Also, two CTs by Huang et al. and Rodrigo et al. showed there is no significant improvement in left ventricular function clinically [14,27]. The study of Rodrigo et al. was underpowered as they had a small number of patients and they used echocardiography rather than magnetic resonance imaging (MRI) to observe the effects on LV [27]. Even though single-photon emission computed tomography (SPECT) imaging showed an improvement in myocardial perfusion after three months of stem cell treatment, since SPECT imaging was not repeated in the control group, the effect of bone marrow-derived mesenchymal stem cells (MSC) therapy on myocardial perfusion can not be evaluated [27].

Two other RCTs focused on patients who received stem cells with coronary artery bypass grafting (CABG) [12,36]. Duan et al. had a treatment group of 24 patients having CABG + BMMNC and a control group of 18 patients who only underwent CABG [12]. One year post-surgery follow-up with echocardiography showed significant improvement in LV function including improvements in the end-diastolic dimension of the left ventricle (LVEDD), end-systolic dimension of the left ventricle (LVESD), LVEDV indexed to body surface area (LVEDVI), LVESV indexed to body surface area (LVESVI), the mass of left ventricle (LV-mass) and LV-mass indexed to body surface area (LV-mass I) compared to the data collected before the operation in CABG+BMMNC group [12]. 

Similarly, Yerebakan et al. had 35 patients who received intramyocardial CD133 (+) bone marrow stem cell transplant + CABG, and 20 patients who only had CABG [36]. Follow-up after 18 months post-surgery showed intramyocardial stem cell therapy was well tolerated but did not have many significant improvements [36]. The authors mentioned that no follow-up angiography was performed, there was an unplanned withdrawal of patients which resulted in incomplete follow-up testing, and a limited number of patients were available for the final analysis [36]. In addition, MRI was not available in the preoperative assessment, so the results of this study should be accepted with caution [36].

Most of the mentioned studies agreed that there was a significant improvement in myocardial function, mainly the left ventricular end-diastolic volume and left ventricular end-systolic volume as well as ejection fraction after treatment with stem cell therapy. In addition, they showed no evidence of adverse effects in patients after receiving stem cell therapy. 

However, some of the included studies reported there was no improvement or benefit from stem cell therapy in myocardial infarction treatment, even though stem cell therapy was safe and well-tolerable to those MI patients. The reason behind it is the studies were not done in a larger population and many patients were lost during follow-up. In addition to that, the optimum time for the administration of stem cells is not yet established.

So, from our systematic review, we can conclude that we need to perform more trials in a larger population and follow up with them closely to find out the effectiveness of stem cell therapy in patients with myocardial infarction.

Types of Stem Cells Used 

One randomized clinical trial (RCT) performed by Gao et al. used Wharton's Jelly-derived mesenchymal stem cells (WJ-MSC) [6]. WJ-MSCs display more cardiovascular differentiation potential and as they are immune privileged, they can be transplanted into unrelated recipients [6].

Another two studies used cardio sphere-derived autologous stem cells [17,18], whereas Naseri et al. in their RCT used CD133 (+) and mononuclear cells [23]. 

In the study of Rodrigo et al. bone marrow-derived mesenchymal stem cells (MSC), a subpopulation of bone marrow cells was used which can differentiate into several cell types including vascular cells, functional cardiomyocytes, etc [27]. In some preclinical models of acute myocardial infarction, it is seen that MSC transplantation promotes neovascularization and myogenesis, which in turn results in improved myocardial function [27].

San Roman et al. studied four groups which include one group of 30 patients receiving bone marrow mononuclear cells (BMMNC), 30 patients assigned to granulocyte colony-stimulating factor (G-CSF), 29 patients receiving G-CSF + BMMNCs, and a placebo group of 31 patients receiving standard therapy [29].

Quyyumi et al. in their RCT in 161 patients used autologous CD34 (+) cell (CLBS10) in 78 patients [26], whereas Peregud-Pogorzelska et al. delivered autologous bone marrow-derived lineage negative stem/progenitor cells in 15 patients in the treatment arm [25]. The researchers of the rest of our 24 included studies used autologous bone marrow mononuclear stem cells to treat the patients. We observed that the type of stem cell used did not have any influence on the outcome of this therapy.

**Figure 3:**
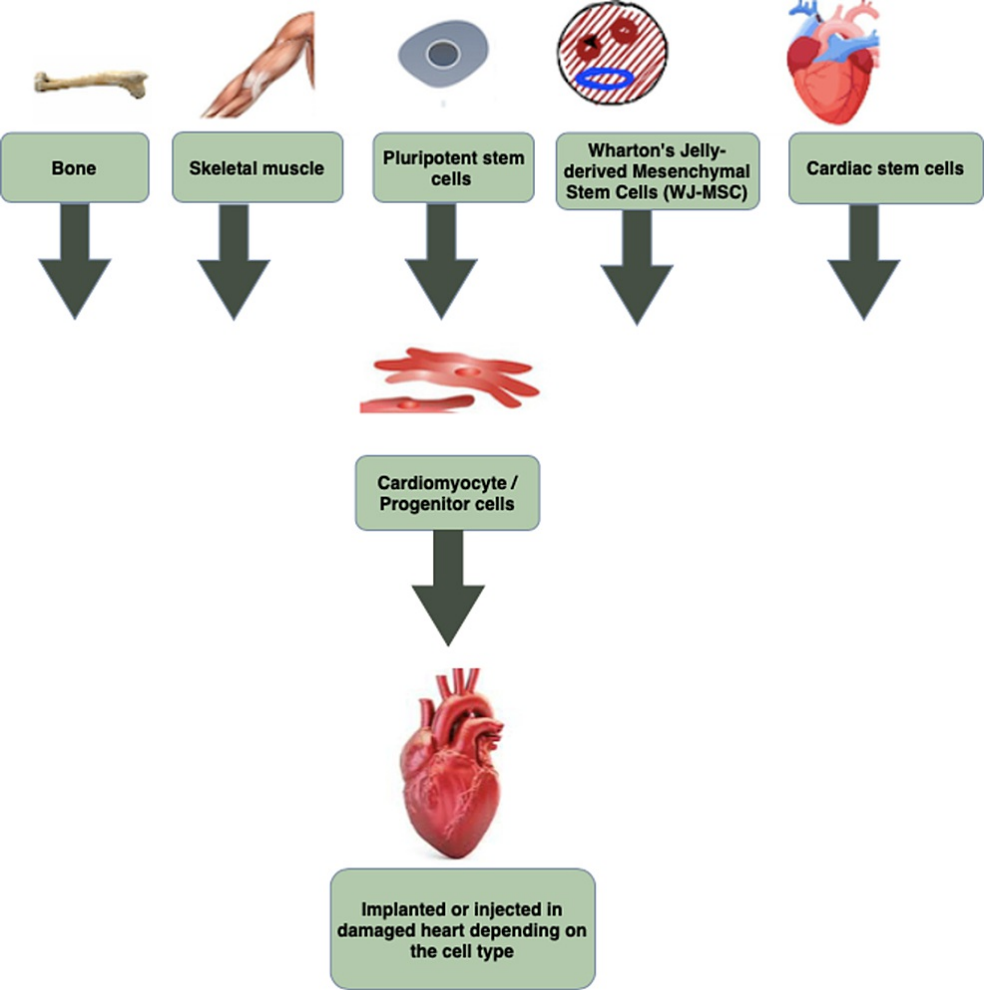
Different types of stem cells used to regenerate the damaged heart muscle

Figure 3 given below illustrates the different types of stem cells used to regenerate the damaged heart muscle. 

Route and Time of Administration of Stem Cell

Regarding the route of administration, intracoronary administration of the stem cell was performed in all our included studies. In addition to that, Moreira et al. in their randomized control trial (RCT) showed retrograde approach (intravenous coronary approach) to deliver stem cells was safe and cell retention by cardiac tissue is more in the anterograde (intra-arterial coronary) approach [21]. Timing of administration did not have much effect on the outcome. Huang et al. in their clinical trial (CT) divided the control group into three subgroups, and they got bone marrow mononuclear stem cells (BMMNC) infusion within two hours, three to seven days after percutaneous coronary intervention (PCI), and seven to thirty days after PCI respectively [14]. Effects of cell therapy given within 24 hours were noticed the same as given three to seven days after the primary PCI [14]. Similarly, Sürder et al. in their RCT demonstrated there is not much difference in the outcomes in groups where BMMNCs are administered five to seven days after ST-elevation myocardial infarction vs in groups where it is administered three to four weeks later [31].

Limitations

There are some limitations of our study, we had a small number of people used in these studies, and a few of them were lost during the follow-up. Additionally, the optimal time of stem cell delivery has not been determined. Moreover, we included only the studies conducted from 2011- 2021 to concentrate more on the updated information.

## Conclusions

Our study focused on evaluating the safety and effectiveness of stem cell therapy in patients with acute myocardial infarction. We found that most of our included studies showed significant improvement in myocardial function after stem cell therapy, but some of the studies failed to show the same improvement. Also, we observed that the stem cell therapy was safe, well-tolerated and no major adverse effects were reported. Because the result was still inconsistent and contradictory, we need to perform high-quality, well-designed clinical trials with a large sample size and more comparable results to assess and establish the efficacy of stem cell therapy in patients with acute myocardial infarction.
